# Expanding medical student interaction in neurology with a redesigned student interest group in neurology (sign) chapter

**DOI:** 10.1186/s12909-021-02641-8

**Published:** 2021-04-17

**Authors:** Rohit Gummi, Ross Smith, Raghav Govindarajan

**Affiliations:** Department of Neurology, University of Missouri-Columbia School of Medicine, Columbia, MO USA

**Keywords:** Neurology, Medical education, Undergraduate curriculum

## Abstract

**Background:**

Student Interest Group in Neurology (SIGN) chapters across the medical schools in the United States provide opportunities for medical students to participate in clinical, research, and service activities in neurology. Despite these, applicants for the field of neurology have traditionally been low.

**Methods:**

Following changes were introduced: an open board style SIGN chapter executive committee with greater active engagement of first and second year students. New activities included journal clubs, hands on workshops, celebration/cause events (example ALS walk). In addition, a free neurology clinic was introduced. Activities were planned in consultation with office of medical education, and were organized during ‘down times’. Data on student enrollment, activities successfully carried out, students interested in neurology residency, number of neurology-related research projects with student involvement were collected prior to changes and compared to values after changes were introduced.

**Results:**

Post intervention, student engagement in neurology activities and projects increased significantly. However, a similar increase in applications to neurology residency was not yet observed.

**Conclusions:**

An open chapter with early engagement and involvement of first and second year medical students, creating a variety of chapter activities with greater hands on involvement, planned in conjunction with office of medical education has reinvigorated our SIGN chapter.

## Background

Student Interest Group in Neurology (SIGN) chapters are interest groups in medical schools across the United States that provide opportunities for medical students to participate in clinical, research, and service activities in neurology. Despite this, the number of students applying into the field of neurology has traditionally been low [1]. Ralph Jozefowicz defined the term ‘neurophobia’ as a fear of the neural sciences and clinical neurology that is due to the students’ inability to apply their knowledge of basic sciences to clinical situations [2]. This is often used to explain medical students’ poor understanding and interest in neurology. It was emphasized that integrating basic science and clinical neuroscience is the key to curing ‘neurophobia.’ Exposure to clinical aspects and the latest treatments in neurology outside the regular medical school curriculum should help students not only better understand neurology, but also make them more likely to consider a career in the field. The students, faculty, and staff working together made efforts to revitalize their local SIGN chapter. With these changes, we aimed to increase interest in the field of neurology and the number of students applying for neurology residency.

## Methods

This study was undertaken at a public university medical school in the United States with 400 to 500 students across four years. The medical school curriculum at this institution is line with other programs in the United States in that it is a four-year program. The first two years are spent with pre-clinical studies. At the end of the second year, the first United States Licensing Examination is taken. The third and fourth year are clinical years, where students spend time on physician led teams and participate in direct patient care. The interventions noted in this study involved students from all levels of study in the school of medicine. We employed 4 major components of change including: expanding the leadership, early engagement of students, scheduling around school requirements, and offering a variety of activities.

### Changes in SIGN executive committee

The SIGN executive committee was re-organized as an open-board style executive committee with greater engagement of first and second year students. The board consisted of primary positions of president, vice president, treasurer, secretary, and medical student committee representative. It was expanded with the addition of preclinical years class representative, community outreach chair, and secondary positions for any of the previously listed positions. Positions exclusively for first and second year students allowed for early involvement of pre-clinical students. Events were delegated among the positions to keep members involved and allowed for a variety of events covering a variety of interests.

#### Early engagement of students with continued involvement

Students were engaged early with events involving faculty and events that overlapped multiple areas of interests. Continued involvement was encouraged through research opportunities, a student run free neurology clinic for the community, and community service events.

For research, a concise summary of research opportunities with faculty within our institution and research programs at outside institutions was provided. This list was sent to the SIGN chapter email group. Another major initiative was a free neurology clinic for uninsured patients which collaborated with the preexisting student run clinic at our institution. The neurology clinic was held once per month and consisted of teams of one pre-clinical student paired with a clinical student who see patients. The students then present to attending and resident neurologists. We placed a special emphasis on allowing the first and second year students to lead the patient encounter as much as they felt comfortable.

#### Activities to Pique Student Interest

A variety of events were held throughout the year to engage students. This first event of the year was an informal event a local restaurant with neurologist representing various sub-specialties. Through the year, interactive labs were held in partnership with local biotechnology companies to provide simulated cases and experiences for students. These included simulations with botulinum toxin injections, lumbar punctures, and interventional neurology simulations. We also had an on-site skills lab to help students learn and participate in an electromyography and nerve conduction studies.

Patient experience panels were also held featuring patients with neurologic disorders who shared their outlook and answered questions about their experience. This was possible by working with the faculty at our institution or neurology clinics in the surrounding areas. The neurologists were able to assess which patients would be interested in speaking with students.

Community service events were held including raising money and awareness for the Amyotrophic Lateral Sclerosis (ALS) foundation by participating in the annual ALS walk. We subsequently helped organized a Myasthenia Gravis (MG) walk. A community outreach event involved interacting with elementary to middle school age children at a local community center with events revolving around the brain.

To aid in preparation for school exams and United States Medical Licensing exams, small group sessions were held involving localization cases and a video review session covering commonly tested principles. We also carried out various journal club discussions pertaining to issues in neurology.

### Scheduling around School Activities

A major barrier of student participation is conflict with other school requirements. As a result, events were scheduled during lunch hours and in the evenings. Our medical school employs an 8-week block system with exams at the end. As a result, the activities were scheduled in the first four weeks to avoid the latter weeks when students focused on exams.

### Data Collection and Analysis

Planned activities were carried out during the 2015–2018 calendar years. The following parameters were compared: student enrollment, activities planned and successfully carried out, Students interested in neurology residency, number of neurology-related research projects with student involvement.

Student enrollment in the SIGN chapter was determined by the students on the email list at the end of the academic year. Students would enroll in the list at the opening meeting or at events through the year. The number of students interested in neurology residency and number of neurology-related research projects were determined by informal polling during the various SIGN events throughout the year. The number of students entering the field of neurology was obtained from official match list released by the medical school. The data was analyzed using linear regression with P value of 0.05 considered significant. We assessed whether or not there was a positive linear relationship between the data over time.

## Results

There was a significant increase in student enrollment, with over 50 % of members being 1st and 2nd year students. There was also a significant increase in students involved in research and students interested in neurology. Initially, fewer than 5 % of the school was engaged with SIGN events. That grew to over 22 % after changes were made (Table 1). The total number of students enrolled ranged from 400 to 424 due to changing class sizes. The SIGN organization carried out many more events with the new structure while involving more neurology faculty members. However, there was not a statistically significant increase in students applying and matching into neurology residencies. From 2014 to 2019, the number of students applying to neurology residency were 2, 1, 4, 2, 2, and 6 (*P* = .197). Although not statistically significant, there was an increase that could be clinically significant. The significant increase in interest in the field has not immediately resulted in significantly higher neurology applicants.

**Table 1:** Comparison of Pre- and Post-Intervention Engagement in Neurology

		Intervention initiated			
	2014–2015	2015–2016	2016–2017	2017–2018	*P* Value
**Student enrollment**	8/400 (2 %)	15/400 (3.75 %)	60/417 (14.39 %)	95/424 (22.41 %)	0.03
**Activities organized**	2	3	8	12	0.02
**Students involved in neuroscience research**	1/400 (0.25 %)	4/400 (1.0 %)	6/417 (1.4 %)	7/424 (1.65 %)	0.03
**Students interested in neurology residency**	1/400 (0.25 %)	4/400 (1.0 %)	8/417 (1.92 %)	14/424 (3.3 %)	0.01

## Discussion

In the United States, the number of students applying for neurology residency (nationally) has remained stable at 2.5 % [1]. However, the American Academy of Neurology (AAN)’s Workforce Task Force found that the US is currently experiencing an 11 % shortfall in the number of neurology needed for patient care will grow to 19 % by 2025 [1]. Given this growing gap in care for neurologic issues, significant changes have been made to the AAN SIGN program in order to identify activities that better engage students [3].

An open chapter with early engagement of students, strategic scheduling of events, and hosting a variety of activities has significantly increased the student and faculty involvement in our SIGN chapter. We hope to encourage other SIGN chapters to implement these changes to increase interest, participation, and ultimately increase the number of students matching into the field of neurology.

At our institution, we found that the class of students with the highest number of students applying to neurology tended to have the highest number interested in neurology before third year of medical school began. We saw that students often did not pick their field as neurology in their third year as we expected, but were already leaning toward this area before their clinical years. Students have often cited general perception of high burnout, lower pay, and fewer treatment options as the reason for avoiding neurology. We are unable to change the general perception of the field, but can increase exposure to the field for younger students so those that are interested do not miss out.

This study was limited by multiple factors including the small sample size and time frame. There was no prior needs assessment for this study and future studies comparing various interventions would provide further valuable information. There was also no have data on other specialties to be able to differentiate trends in neurology with overall trends in the school such as research involvement.

## Conclusions

The interventions made to the SIGN chapter at our institution has increased exposure to neurology to a significant amount of medical student. Having a higher number of students enter their clerkship years with previous exposure to neurology led to much higher engagement. We ultimately hoped to note an increase in students entering the field of neurology. A significant increase in neurology applicants was not immediately noted in our study; however, we did see an increase that might be significant over a longer time frame.

## Data Availability

All data generated or analyzed during this study are included in this published article.

## Abbreviations

SIGN: Student Interest Group in Neurology.
EMG: Electromyography.
ALS: Amyotrophic Lateral Sclerosis.
MG: Myasthenia Gravis.
AAN: American Academy of Neurology.
